# Circulating tumor DNA refines consolidation immunotherapy for limited-stage small cell lung cancer patients

**DOI:** 10.1038/s41392-025-02445-y

**Published:** 2025-10-16

**Authors:** Yin Yang, Yuqi Wu, Jingjing Zhao, Tao Zhang, Kailun Fei, Xiaotian Zhao, Lei Deng, Zhihui Zhang, Ying Jiang, Jianyang Wang, Wenyang Liu, Xin Wang, Song Wang, Hua Bao, Xue Wu, Minyi Zhu, Qiuxiang Ou, Wei Tang, Luhua Wang, Zhijie Wang, Nan Bi

**Affiliations:** 1 Department of Radiation Oncology, National Cancer Center/National Clinical Research Center for Cancer/Cancer Hospital, Chinese Academy of Medical Sciences and Peking Union Medical College, Beijing, China; 2 State Key Laboratory of Molecular Oncology, CAMS Key Laboratory of Translational Research on Lung Cancer, Department of Medical Oncology, National Cancer Center/National Clinical Research Center for Cancer/Cancer Hospital, Chinese Academy of Medical Sciences and Peking Union Medical College, Beijing, China; 3 Geneseeq Research Institution, Nanjing Geneseeq Technology Inc., Nanjing, China; 4 Department of Radiology, National Cancer Center/National Clinical Research Center for Cancer/Cancer Hospital, Chinese Academy of Medical Sciences and Peking Union Medical College, Beijing, China; 5 Department of Radiation Oncology, National Cancer Center/National Clinical Research Center for Cancer/Cancer Hospital & Shenzhen Hospital, Chinese Academy of Medical Sciences and Peking Union Medical College, Shenzhen, China; 6 State Key Laboratory of Molecular Oncology, Department of Radiation Oncology, National Cancer Center/National Clinical Research Center for Cancer/Cancer Hospital, Chinese Academy of Medical Sciences and Peking Union Medical College, Beijing, China

**Keywords:** Predictive markers, Prognostic markers

## Abstract

Despite the lack of predictive biomarkers and a prognostic stratification strategy, immune checkpoint inhibitor (ICI) has shown promise in improving outcomes for patients with limited-stage small cell lung cancer (LS-SCLC). We evaluated the potential of circulating tumor DNA (ctDNA) to dynamically predict outcomes in patients with LS-SCLC receiving concurrent chemoradiotherapy (CCRT) with or without consolidation ICI. We analyzed 490 serial samples collected from 144 LS-SCLC patients at baseline (t0), post-induction chemotherapy and pre-thoracic radiotherapy (t1), post-radiotherapy (t2), and progressive disease (t3). For 44 patients receiving consolidation ICI with serplulimab, an investigational PD-1 inhibitor, ctDNA dynamics during consolidation ICI were also assessed at multiple time points. Patients with undetectable ctDNA after CCRT had good outcomes with or without consolidation ICI, whereas ctDNA-positive patients at t2, indicating poor response to CCRT, derived survival benefit from consolidation ICI. Notably, ctDNA status at t1 appeared more predictive than at t2. A three-level risk stratification strategy integrating t1 ctDNA status with radiological tumor shrinkage identified a high-risk subgroup of patients who achieved significantly improved progression-free survival (PFS) (hazard ratio [HR], 0.24; 95% confidence interval [CI], 0.08–0.75; *p* = 0.014) and overall survival (OS) (HR, 0.06; 95% CI, 0.00–0.42; *p* = 0.001) from consolidation ICI, prioritizing CCRT plus consolidation ICI. Furthermore, maintaining ctDNA negativity during consolidation ICI was associated with favorable outcomes. These data provide valuable insights into the individualized management of LS-SCLC in the era of immunotherapy.

## Introduction

Small cell lung cancer (SCLC) is a highly aggressive malignancy that accounts for 15–20% of newly diagnosed lung cancer cases.^1^ Approximately one-third of SCLC cases are classified as limited-stage SCLC (LS-SCLC), and concurrent platinum-based chemoradiotherapy (CCRT) with or without prophylactic cranial irradiation (PCI) used to be the standard of care. However, many patients progressed and exhibited a poor prognosis, with a median survival duration of 25–30 months, despite initial therapeutic responses.^2^

The phase III ADRIATIC trial recently demonstrated that consolidation durvalumab after CCRT significantly improved progression-free survival (PFS) and overall survival (OS) compared with CCRT alone,^3^ establishing it as a new standard of care for patients with LS-SCLC. However, the addition of durvalumab only achieves 12% and 8.9% improvement in 2-year PFS and OS rates, respectively. Moreover, approximately 25% of LS-SCLC patients can be cured by CCRT alone and are unnecessarily exposed to the toxicities of consolidation immunotherapy,^3,4^ suggesting the potential for a stratification strategy. Serplulimab, a PD-1 inhibitor, combined with chemotherapy demonstrated significant antitumor activity in the phase III ASTRUM-005 trial and has been recommended as a first-line treatment for extensive-stage SCLC (ES-SCLC) in China.^5^ The efficacy and safety of serplulimab in LS-SCLC patients are currently under evaluation in an ongoing phase II trial of consolidation serplulimab following CCRT.^6^

The prevailing predictive biomarkers for immunotherapy, mainly PD-L1 expression,^7^ tumor mutational burden (TMB),^8^ and molecular subtypes (SCLC-A, -N, -P, and -I),^9,10^ are far from satisfactory for SCLC. The exploration of biomarkers predictive of benefit from consolidation immune checkpoint inhibitor (ICI) is urgently needed to guide personalized decision making in the context of precision medicine.

Plasma circulating tumor DNA (ctDNA) is a promising noninvasive tool for detecting residual disease, assessing treatment response, tracking disease progression, personalizing therapeutic strategies, and investigating resistance mechanisms in non-small cell lung cancer.^11–15^ Recent studies have shown that the integration of ctDNA analysis and radiological assessments could predict clinical outcomes more accurately^16^ and guide ICI therapy selection.^17^

Herein, we conducted a longitudinal ctDNA analysis integrating patient data from two clinical trials evaluating treatment regimens for LS-SCLC: one assessing CCRT alone and the other evaluating CCRT followed by serplulimab. The aim of this study was to evaluate dynamic changes in ctDNA and its integration with radiological tumor shrinkage to predict clinical outcomes, identify patients who may benefit from consolidation ICI therapy after CCRT, and monitor treatment efficacy in LS-SCLC patients.

## Results

### Patients and samples

A total of 100 patients who received CCRT alone and 44 patients who received consolidation ICI were included. The clinical characteristics of the patients are summarized in Table 1. Among the 144 patients, the majority were male (77.4%), were current/former smokers (66.0%), and had stage III disease at diagnosis (89.6%). As of April 20, 2025, disease progression had occurred in 75 patients (52.1%, 75/144), with 57 patients in the CCRT-only group and 18 patients in the consolidation ICI group. Similarly, 48 patients (33.3%, 48/144) died, with 41 and 7 patients in the CCRT-only and consolidation ICI groups, respectively. The median follow-up duration was 37.8 months (interquartile range [IQR], 33.3–44.5) for the CCRT-only group and 30.1 months (IQR, 25.5–32.1) for the ICI group.

**Table 1:** Clinical characteristics of patients with limited-stage small cell lung cancer

Characteristics	Overall (*n* = 144)	ICI (*n* = 44)	CCRT-only (*n* = 100)	*p*
Age group, No. (%)				0.787
<60	81 (56.2)	24 (54.5)	57 (57.0)	
≥60	63 (43.8)	20 (45.5)	43 (43.0)	
Sex, No. (%)				0.057
Male	137 (77.4)	29 (65.9)	82 (82.0)	
Female	40 (22.6)	23 (29.9)	18 (18.0)	
Smoking, No. (%)				0.178
Yes	95 (66.0)	25 (56.8)	70 (70.0)	
No	49 (34.0)	19 (43.2)	30 (30.0)	
ECOG PS, No. (%)				0.443
0	48 (33.3)	17 (38.6)	31 (31.0)	
1	96 (66.7)	27 (61.4)	69 (69.0)	
T stage, No. (%)				0.966
T1	29 (20.1)	9 (20.5)	20 (20.0)	
T2	50 (34.7)	14 (31.8)	36 (36.0)	
T3	27 (18.8)	9 (20.5)	18 (18.0)	
T4	38 (26.4)	12 (27.3)	26 (26.0)	
N stage, No. (%)				0.410
N0	3 (2.1)	2 (4.5)	1 (1.0)	
N1	19 (13.2)	5 (11.4)	14 (14.0)	
N2	73 (50.7)	20 (45.5)	53 (53.0)	
N3	49 (34.0)	17 (38.6)	32 (32.0)	
Clinical stage, No. (%)				0.775
II	15 (10.4)	5 (11.4)	10 (10.0)	
III	129 (89.6)	39 (88.6)	90 (90.0)	

*ICI* immune checkpoint inhibitor, *CCRT* concurrent chemoradiotherapy, *ECOG PS* Eastern Cooperative Oncology Group Performance Scale

**Fig. 1:**
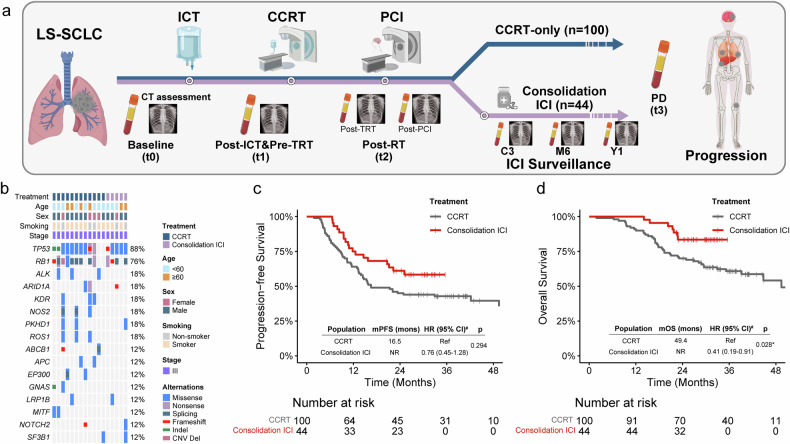
Study design, baseline mutational landscape, and consolidation ICI efficacy. **a** The inclusion of patients with LS-SCLC and the collection of peripheral blood samples at key time points, including baseline (t0), post-ICT & pre-TRT (t1), post-RT (t2), during consolidation ICI (C3, M6, and Y1), and PD (t3). **b** Heatmap summarizing genetic alterations detected in ≥2 patients with baseline (t0) ctDNA samples. **c**, **d** Kaplan‒Meier curves of PFS (**c**) and OS (**d**) for patients treated with CCRT alone and those treated with consolidation ICI. ICI immune checkpoint inhibitor, LS-SCLC limited-stage small cell lung cancer, ICT induction chemotherapy, TRT thoracic radiotherapy, RT radiotherapy, CCRT concurrent chemoradiotherapy, PCI prophylactic cranial irradiation, PD progressive disease, ctDNA circulating tumor DNA, CNV copy number variant, PFS progression-free survival, OS overall survival, HR hazard ratio, CI confidence interval, NR not reached. ^#^HR, 95% CI, and *p* values were estimated using time-dependent Cox regression models. Asterisks indicate levels of statistical significance: *p < 0.05

A total of 490 plasma ctDNA samples were sequenced, comprising 251 samples from the CCRT-only group and 239 samples from the ICI group. These samples were collected at the following time points: 17 at baseline (t0), 144 after induction chemotherapy (ICT) but before thoracic radiotherapy (TRT) (post-ICT & pre-TRT, t1), 194 after completion of radiotherapy (including 114 post-TRT and 80 post-PCI, t2), 41 at progressive disease (PD, t3), 39 at the initiation of cycle 3 (C3), 29 at month 6 (M6), and 26 at year 1 (Y1) of ICI treatment (Fig. 1a). All t0 samples were ctDNA positive, with *TP53* and *RB1* being the most frequently altered genes, detected in 88 and 76% of patients, respectively (Fig. 1b). High concordance was observed between post-TRT and post-PCI samples (agreement: 88%; kappa coefficient: 0.65), supporting their interchangeability (Supplementary Fig. 1). Consequently, t2 ctDNA positivity was defined as positive detection in either post-TRT or post-PCI samples.

### Efficacy of consolidation ICI in patients with LS-SCLC

Compared with 100 patients who received CCRT alone, 44 patients who received consolidation ICI had longer median PFS (16.5 months vs. not reached [NR], Fig. 1c) and OS (49.4 months vs. NR, Fig. 1d). Given the potential for immortal time bias,^18,19^ time-dependent Cox regression models, which treat consolidation ICI as a time-dependent covariate, were applied, revealing that consolidation ICI was associated with a trend toward improved PFS (hazard ratio [HR], 0.76; 95% confidence interval [CI], 0.45–1.28; *p* = 0.294; Fig. 1c) and significantly superior OS (HR, 0.41; 95% CI, 0.19–0.91; *p* = 0.028; Fig. 1d) in LS-SCLC patients.

### Both t1 and t2 ctDNA detection predict an inferior prognosis in LS-SCLC patients receiving CCRT alone

**Fig. 2:**
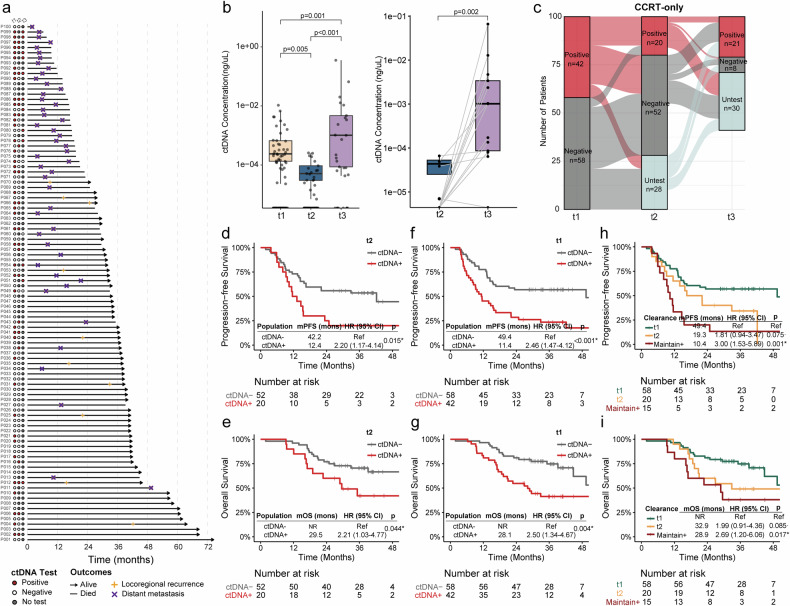
Prognostic values of t1 and t2 ctDNA in LS-SCLC patients receiving CCRT alone. **a** Swimmer plot of 100 patients who received CCRT alone. **b** ctDNA concentrations from t1 to t3 and comparisons of paired t2 and t3 samples. **c** ctDNA positivity dynamics from t1 to t3. **d**, **e** t2 ctDNA detection predicted worse PFS (**d**) and OS (**e**) in CCRT-only patients. **f**, **g** t1 ctDNA detection predicted worse PFS (**f**) and OS (**g**) in CCRT-only patients. **h**, **i** Kaplan‒Meier curves of PFS (**h**) and OS (**i**) in CCRT-only patients stratified by ctDNA clearance status. ctDNA circulating tumor DNA, LS-SCLC limited-stage small cell lung cancer, CCRT concurrent chemoradiotherapy, PFS progression-free survival, OS overall survival, HR hazard ratio, CI confidence interval, NR not reached. Dots denote trend-level significance: ·*p* < 0.1. Asterisks indicate levels of statistical significance: *p < 0.05

The swimmer plot summarizes the ctDNA detection and clinical outcomes of 100 CCRT-only patients (Fig. 2a). In the CCRT-only cohort, ctDNA concentrations significantly decreased from t1 to t2 (*p* = 0.005) but peaked at t3 (*p* < 0.001), with a similar pattern observed in paired t2 and t3 samples (*p* = 0.002) (Fig. 2b), suggesting an association between radiographic progression and ctDNA dynamics as measured by next-generation sequencing. Consistently, mean variant allele frequencies (VAFs) also declined from t1 to t2 (*p* = 0.004) but increased at t3 (*p* < 0.001), with a comparable difference observed in paired t2 and t3 samples (*p* < 0.001) (Supplementary Fig. 2a). The ctDNA detection dynamics tended toward decreased ctDNA detection rates from t1 to t2 (42.0% vs. 27.8%, p = 0.055) (Fig. 2c). A positive t2 ctDNA status, indicative of residual tumor presence after completing CCRT, predicted inferior PFS (HR, 2.20; 95% CI, 1.17–4.14; *p* = 0.015) (Fig. 2d) and OS (HR, 2.21; 95% CI, 1.03–4.77; *p* = 0.044) (Fig. 2e). Similarly, t1 ctDNA detection was also associated with worse PFS (HR, 2.46; 95% CI, 1.47–4.12; *p* < 0.001) (Fig. 2f) and OS (HR, 2.50; 95% CI, 1.34–4.67; *p* = 0.004) (Fig. 2g).

Given that all available t0 samples were ctDNA positive, we tentatively defined patients with negative t1 ctDNA as having achieved early ctDNA clearance at t1. Among CCRT-only patients, a trend toward inferior PFS was observed in 20 patients who achieved delayed ctDNA clearance at t2 compared with patients with early ctDNA clearance at t1 (HR, 1.81; 95% CI, 0.94–3.47; *p* = 0.075) (Fig. 2h). There were 15 patients with persistent ctDNA positivity from t1 to t2, who exhibited significantly poorer PFS than those with t1 clearance (HR, 3.00; 95% CI, 1.53–5.89; *p* = 0.001). Similar results were observed for the OS data (t2 clearance vs. t1 clearance: HR, 1.99; 95% CI, 0.91–4.36; *p* = 0.085; maintain positive vs. t1 clearance: HR, 2.69; 95% CI, 1.20–6.06; *p* = 0.017) (Fig. 2i).

### Identification of the benefit of ICI consolidation via ctDNA detection

**Fig. 3:**
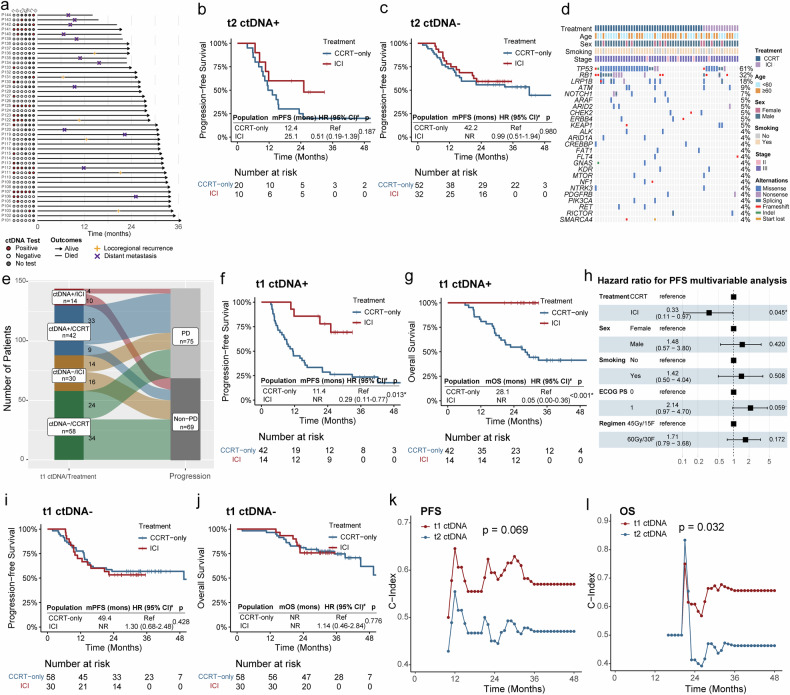
Association between ctDNA detection and clinical outcomes under consolidation ICI therapy. **a** Swimmer plot of 44 patients receiving consolidation ICI. **b** Improved PFS observed in t2 ctDNA-positive patients receiving consolidation ICI compared with patients receiving CCRT alone. **c** Comparison of PFS between t2 ctDNA-negative patients receiving consolidation ICI and patients receiving CCRT alone. **d** Heatmap summarizing genetic alterations detected in ≥2 ctDNA-positive patients at the t1 time point. **e** Association between t1 ctDNA status, treatment, and clinical outcomes. **f**, **g** Improved PFS (**f**) and OS (**g**) were observed in t1 ctDNA-positive patients receiving consolidation ICI compared with patients receiving CCRT alone. **h** Forest plot showing the multivariate time-dependent Cox regression model adjusted for treatment, sex, smoking status, ECOG performance score, and TRT regimen. **i**, **j** Comparison of PFS (**i**) and OS (**j**) between t1 ctDNA-negative patients receiving consolidation ICI and patients receiving CCRT alone. **k**, **l** Comparison of the predictive values of t1 and t2 ctDNA for PFS (**k**) and OS (**l**) in patients receiving consolidation ICI therapy. ctDNA circulating tumor DNA, ICI immune checkpoint inhibitor, CCRT concurrent chemoradiotherapy, PFS progression-free survival, OS overall survival, HR hazard ratio, CI confidence interval, NR not reached, PD progressive disease, ECOG PS Eastern Cooperative Oncology Group Performance Scale, TRT thoracic radiotherapy. ^#^HR, 95% CI, and *p* values were estimated using time-dependent Cox regression models. Dots denote trend-level significance: ·*p* < 0.1. Asterisks indicate levels of statistical significance: *p < 0.05

Swimmer plots depict the length of follow-up, events, and ctDNA positivity (Fig. 3a). Given that t2 represents the latest time point before consolidation ICI initiation, we evaluated its potential as a predictive biomarker. Among 30 t2 ctDNA-positive patients, a trend toward superior PFS was observed in patients receiving consolidation ICI (HR, 0.51; 95% CI, 0.19–1.39; *p* = 0.187), whereas t2 ctDNA-negative patients receiving consolidation ICI or CCRT alone showed comparable PFS (HR, 0.99; 95% CI, 0.51–1.94; *p* = 0.980), suggesting the potential of t2 ctDNA as a predictive biomarker for consolidation ICI (Fig. 3b, c). Similar trends were observed for OS analysis (Supplementary Fig. 3a, b).

Since consolidation ICI significantly reduces the risk of extrathoracic metastasis and early ctDNA dynamic changes can predict chemotherapy outcomes in patients with SCLC,^20,21^ we further investigated whether t1 ctDNA detection could predict the benefit of ICI consolidation at an earlier time point. Among the 144 t1 samples, 56 (39%) were ctDNA positive. Consistent with t0, *TP53* remained the most commonly mutated gene (61%), followed by *RB1* (32%), although no copy number variation (CNV) events were detected in ≥2 patients in this subset (Fig. 3d). Instead, a single start-lost mutation in *SMARCA4* was observed, highlighting differences in mutational profiles between time points. Among ctDNA-positive patients at t1, those who received consolidation ICI presented a significantly lower progression rate than those treated with CCRT alone (chi-square test, *p* < 0.001) (Fig. 3e). Time-to-event analysis consistently demonstrated similar findings for both PFS and OS (Fig. 3f, g). Specifically, t1 ctDNA-positive patients had longer PFS with consolidation ICI than with CCRT-only (median PFS, NR vs. 11.4 months). Time-dependent Cox regression, accounting for immortal time bias, revealed a significant association between consolidation ICI therapy and improved PFS in t1 ctDNA-positive patients (HR, 0.29; 95% CI, 0.11–0.77; *p* = 0.013). In addition, among t1 ctDNA-positive patients, those receiving consolidation ICI demonstrated significantly prolonged OS compared with those treated with CCRT alone (median OS, NR vs. 28.1 months; HR, 0.05; 95% CI, 0.00–0.36; *p* < 0.001). Univariate time-dependent Cox regression analysis identified several potential prognosis-related factors (Supplementary Fig. 4). The multivariate analysis further demonstrated that consolidation ICI were independently associated with improved PFS (HR, 0.33; 95% CI, 0.11–0.97; *p* = 0.045) and OS (HR, 0.07; 95% CI, 0.00–0.61; *p* = 0.010) in t1 ctDNA-positive patients (Fig. 3h; Supplementary Fig. 5). In contrast, among t1 ctDNA-negative patients, consolidation ICI therapy did not significantly improve either PFS (HR, 1.30; 95% CI, 0.68–2.48; *p* = 0.428) or OS (HR, 1.14; 95% CI, 0.46–2.84; *p* = 0.776) compared with those in patients receiving CCRT alone (Fig. 3i, j). Furthermore, t1 ctDNA demonstrated a higher C-index for PFS and OS prediction than that of t2 ctDNA in patients receiving consolidation ICI (Fig. 3k, l), supporting its key role in treatment decision-making and efficacy prediction.

We next explored recurrence patterns stratified by ctDNA dynamics and treatment modality. Among patients who achieved t2 clearance (ctDNA positive at t1 but ctDNA negative at t2), distant metastasis remained the predominant failure pattern in the CCRT-only group, whereas the majority of patients in the ICI group remained relapse free (*p* = 0.022) (Supplementary Fig. 6). These findings suggest that patients who achieve t2 clearance derive significant benefit from consolidation ICI.

### Prognosis stratification by integrating t1 ctDNA with radiological tumor shrinkage

**Fig. 4:**
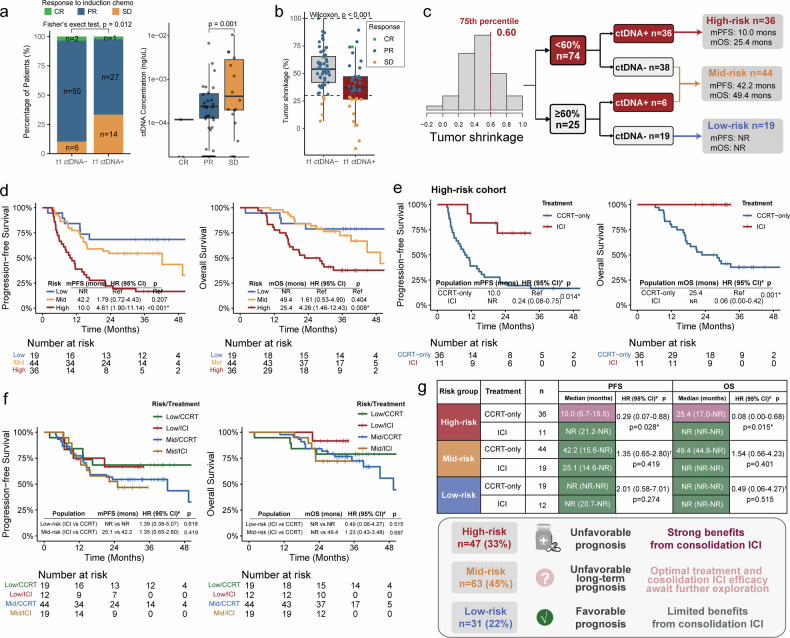
Prognosis stratification by integrating t1 ctDNA with radiological tumor shrinkage. **a** Association of the response to ICT with t1 ctDNA detection and concentration. **b** Association of tumor shrinkage after ICT and t1 ctDNA detection. **c** Three-level risk classification strategy integrating tumor shrinkage and t1 ctDNA detection. **d** Kaplan‒Meier curves of PFS and OS for patients classified into low-, mid-, and high-risk cohorts. **e** There were strong survival benefits under consolidation ICI therapy compared with CCRT alone in high-risk patients. **f** Limited survival benefits under consolidation ICI therapy compared with CCRT alone in low- and mid-risk patients. **g** The number of patients in each subgroup, median survival time, and efficacy of consolidation ICI were estimated using multivariable time-dependent Cox regression adjusted for potential confounding effects. ctDNA circulating tumor DNA, ICT induction chemotherapy, CR complete response, PR partial response, SD stable disease, PFS progression-free survival, OS overall survival, HR hazard ratio, CI confidence interval, ICI immune checkpoint inhibitor, CCRT concurrent chemoradiotherapy, NR not reached. ^#^HR, 95% CI, and *p* values were estimated using time-dependent Cox regression models. ^†^HR, 95% CI, and *p* values were estimated using univariate time-dependent Cox regression analysis, as no variable had *p* values < 0.1 in the univariate analysis. Asterisks indicate levels of statistical significance: *p < 0.05

No patients presented with PD at t1. As shown in Fig. 4a, t1 ctDNA positivity was significantly associated with stable disease (SD) rather than partial or complete response (PR/CR) (Fisher’s exact test, *p* = 0.012). In addition, ctDNA concentrations were significantly higher in patients with SD than in those with PR (*p* = 0.001). Consistently, ctDNA-positive patients presented a decreased percentage of tumor shrinkage at t1 (*p* < 0.001, Fig. 4b). These findings suggest that t1 ctDNA can reflect residual tumor burden, supporting a prognostic stratification strategy combining ctDNA detection with radiological response (Supplementary Fig. 7a, b). Thus, a prognostic stratification model was developed for CCRT-only patients (Supplementary Fig. 7c). Fifty-two double responders (both radiological and molecular) to ICT had better prognosis than 34 partial responders (either radiological or molecular), whereas the 14 nonresponders (neither radiological nor molecular) had the poorest prognosis (Supplementary Fig. 7d, e).

To identify patients with a prolonged prognosis under CCRT, a tumor shrinkage threshold of 60% (75^th^ percentile) was further applied (Fig. 4c). The low-risk cohort comprised 19 patients with tumor shrinkage ≥60% and a t1 ctDNA-negative status, with high 4-year PFS and OS rates of 68.4% and 78.9%, respectively (Fig. 4d). The mid-risk group included 38 patients with tumor shrinkage <60% and t1 ctDNA-negative status and 6 patients with tumor shrinkage ≥60% and t1 ctDNA-positive status. Patients with tumor shrinkage <60% and a t1 ctDNA-positive status were classified as high risk. The mid-risk group exhibited lower 2- and 4-year PFS rates than the low-risk group (2-year, 54.5% vs. 68.4%; 4-year, 43.6% vs. 68.4%). High-risk patients had significantly worse PFS (HR, 4.61; 95% CI, 1.90–11.14; *p* < 0.001) and OS (HR, 4.26; 95% CI, 1.46–12.43; *p* = 0.008) than low-risk patients (Fig. 4d).

Furthermore, in high-risk patients, significantly improved PFS (HR, 0.24; 95% CI, 0.08–0.75; *p* = 0.014) and OS (HR, 0.06; 95% CI, 0.00–0.42; *p* = 0.001) were observed in those treated with consolidation ICI than in those receiving CCRT alone (Fig. 4e). However, limited short-term benefits were observed in mid- and low-risk patients (Fig. 4f). To determine independent prognostic significance, variables with *p* values < 0.1 in the univariate time-dependent Cox analysis were included in a multivariate model adjusted for potential confounders (Fig. 4g; Supplementary Fig. 8). In the high-risk group, which exhibited a poor prognosis under CCRT, consolidation ICI therapy provided significant benefits. In contrast, the low-risk group, with a favorable prognosis under CCRT, showed limited benefits from consolidation ICI. For the mid-risk group, which had an acceptable short-term prognosis but a poor long-term prognosis under CCRT, limited short-term benefits were detected. However, the potential long-term benefits remain unclear due to the moderate follow-up duration. The optimal treatment and the efficacy of consolidation ICI require further investigation.

### ctDNA surveillance during consolidation ICI predicts clinical outcome

**Fig. 5:**
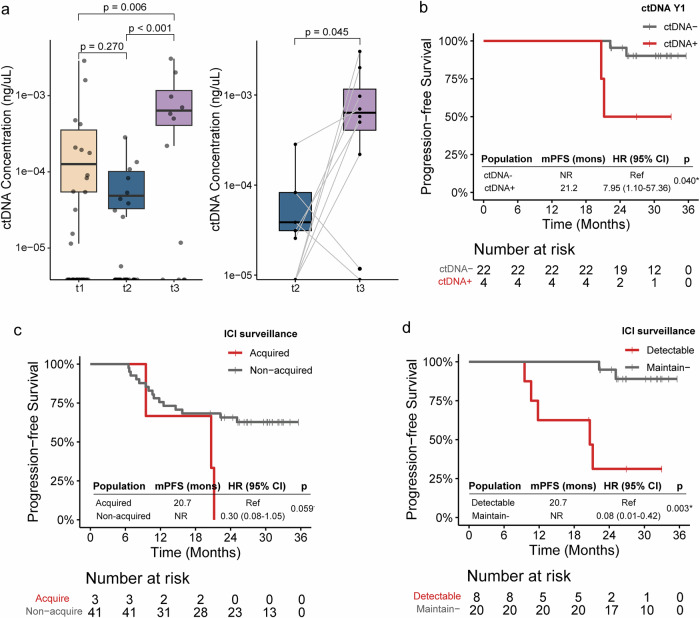
ctDNA surveillance in patients receiving consolidation ICI. **a** ctDNA concentrations from t1 to t3 and comparisons of paired t2 and t3 samples. **b** Kaplan‒Meier curves of PFS for patients with positive and negative ctDNA status at Y1. **c** Kaplan‒Meier curves of PFS for patients with and without acquired ctDNA detection during ICI surveillance. **d** Kaplan‒Meier curves of PFS for patients who maintained a negative ctDNA status and had detectable ctDNA at ≥1 time point during ICI surveillance. ctDNA circulating tumor DNA, ICI immune checkpoint inhibitor, PFS progression-free survival, Y1 year 1, HR hazard ratio, CI confidence interval, NR not reached. Dots denote trend-level significance: ·*p* < 0.1. Asterisks indicate levels of statistical significance: *p < 0.05

In addition to serial samples collected at t1, t2, and t3, the consolidation ICI cohort underwent additional ctDNA surveillance prior to progression during ICI treatment, including samples collected at C3, M6, and Y1 (Fig. 3a). Similar to the CCRT-only cohort, patients in the consolidation ICI cohort presented a decrease in both ctDNA concentrations and mean VAFs after RT, followed by a peak at the time of progression, with consistent trends observed in the matched t2 and t3 samples (Fig. 5a; Supplementary Fig. 2b). Among the six patients with ctDNA-positive status at t2 and paired C3 samples, one (16.7%) achieved ctDNA clearance at C3 and remained alive and progression free after 33.0 months of follow-up. The ctDNA detection rates and concentrations did not change significantly during consolidation ICI treatment. Notably, ctDNA detection at later surveillance time points appeared to predict worse PFS, especially at Y1 (HR, 7.95; 95% CI, 1.10–57.36; *p* = 0.040) (Fig. 5b). During surveillance, ctDNA was newly detected in three patients, one at C3 and two at Y1, all of whom developed disease progression within two years. Compared with patients with acquired ctDNA detection, those without acquired ctDNA detection exhibited better PFS (HR, 0.30; 95% CI, 0.08–1.05; *p* = 0.059) (Fig. 5c). In addition, among the 28 patients with serial samples between C3 and Y1, those with persistently negative ctDNA (*n* = 20) exhibited prolonged PFS compared with those with at least one detectable ctDNA sample (HR, 0.08; 95% CI, 0.01–0.42; *p* = 0.003) (Fig. 5d). However, due to the limited sample sizes within each subgroup, these findings should be interpreted with caution and warrant further validation in larger prospective cohorts.

## Discussion

In the era of immunotherapy and precision oncology, there is an unmet need to develop real-time, minimally invasive biomarkers as well as stratification strategies to capture therapeutic response and guide clinical decision making. To the best of our knowledge, this is the first study exploring the utility of ctDNA monitoring for patients with LS-SCLC receiving CCRT with or without consolidation ICI. We determined the prognostic and predictive value of early ctDNA status after ICT, with further incorporation of t1 ctDNA status with radiological assessment to establish a ctDNA-based prognostic stratification strategy. This approach distinguishes patients with different prognoses and identifies those most likely to benefit from consolidation ICI therapy.

Currently, multiple prognostic and predictive biomarkers, including TMB, PD-L1 expression, and molecular subtypes, have been explored in SCLC.^7–9,22,23^ For example, Hellmann et al. reported that high TMB was associated with improved PFS in patients with relapsed SCLC receiving nivolumab ± ipilimumab.^22^ However, Horn et al., through a subgroup analysis of the IMpower133 trial in ES-SCLC, reported no clear predictive benefit of the TMB for atezolizumab vs. placebo, highlighting uncertainty regarding the optimal TMB cutoff.^8^ Similarly, Reinmuth et al. analyzed 21 ES-SCLC patients receiving durvalumab plus tremelimumab and reported no significant difference in OS between patients with PD-L1 expression ≥1% and those with <1%.^23^ Likewise, Park et al. reported comparable survival outcomes in LS-SCLC patients treated with concurrent chemoradiotherapy plus durvalumab regardless of PD-L1 status.^7^ Molecularly, the SCLC-I subtype is associated with an inflammatory TME characterized by increased immune activation, which may underlie its greater benefit from the addition of immunotherapy to chemotherapy.^9^ In contrast to these conventional biomarkers, ctDNA monitoring has emerged as a promising dynamic biomarker, which has been studied primarily in ES-SCLC patients.^21,23–25^ Murciano-Goroff et al. reported that patients who experienced a >2-fold decrease in ctDNA concentration on cycle 2 day 1 were sensitive to platinum-based therapy, resulting in longer PFS and OS.^21^ Reinmuth et al. demonstrated that reductions in on-treatment ctDNA levels were correlated with longer OS in platinum-refractory ES-SCLC patients treated with durvalumab plus tremelimumab.^23^ Han et al. reported that low on-treatment blood TMB (bTMB) was associated with longer PFS and OS in relapsed ES-SCLC patients receiving anlotinib, sintilimab, and chemotherapy.^24^ Additionally, sustained molecular response, defined by persistent elimination of the cell-free tumor load (cfTL) before and after treatment, is associated with a significantly improved prognosis in patients with metastatic SCLC.^25^ Despite growing interest in biomarker research and ctDNA monitoring, most studies have focused on ES-SCLC, with limited data available for LS-SCLC. The recent ADRIATIC trial,^3^ which demonstrated the clinical benefits of consolidation durvalumab after chemoradiotherapy in LS-SCLC patients, underscores the urgent need to identify reliable biomarkers in LS-SCLC patients to better guide patient selection and personalize therapeutic strategies.

We determined the prognostic value of ctDNA at multiple time points after ICT initiation and revealed that molecular responders to platinum-based chemotherapy or thoracic radiotherapy had favorable survival outcomes. Notably, in our study, TRT was administered concurrently with the third cycle of chemotherapy, which has been shown to offer comparable efficacy and favorable toxicity to TRT starting with the first cycle of chemotherapy^26^ and is widely used in other studies.^7,27^ Moreover, we found high concordance in ctDNA status between the post-TRT and post-PCI time points, suggesting a single post-radiation assessment time point; this may be because PCI is typically administered shortly after TRT to reduce the risk of brain metastasis, where peripheral ctDNA detection is generally considered less sensitive for brain metastasis detection.^28,29^ Additionally, our results, together with those of previously reported studies, revealed the limited prognostic value of baseline ctDNA because of the high detection rate (over 90%) in treatment-naïve LS-SCLC patients.^30,31^

In our study, we demonstrated that detectable ctDNA at the early t1 time point served as a strong predictor of optimal efficacy for consolidation ICI therapy. Given that distant metastasis is the predominant failure pattern in LS-SCLC patients treated with CCRT,^32,33^ consolidation ICI plays a critical role in enhancing immune surveillance against micrometastatic disease, ultimately reducing the risk of distant metastasis.^20,34^ Our findings revealed that a subset of molecularly poor responders to chemotherapy remain at high risk of distant metastasis despite achieving ctDNA clearance following CCRT (t2 clearance). These patients derive significant benefit from consolidation ICI therapy. Therefore, ctDNA assessment at the earlier t1 time point allows for a more accurate identification of high-risk patients who will benefit substantially from consolidation ICI therapy. In other words, if patient stratification was based solely on ctDNA status at t2, individuals who were ctDNA positive at t1 but negative at t2 would be misclassified as responders. This misclassification would lead to an underestimation of their risk and missed opportunities for effective consolidation ICI. Furthermore, our results underscore that ICI provides an effective systemic therapeutic alternative for LS-SCLC patients with suboptimal chemotherapy response, highlighting the importance of integrating early molecular response evaluation into treatment decision-making. By integrating ctDNA status with radiological tumor shrinkage at t1, we developed a refined risk stratification strategy, classifying patients into three subgroups with distinct clinical trajectories and treatment recommendations. Patients in the low-risk subgroup had an OS (4-year OS rate of 78.9%) similar to that of patients with stage I operable SCLC,^35^ suggesting that this subgroup has a high possibility of cure after definitive CCRT alone. In the high-risk subgroup, patients with favorable features derived significant benefit from consolidation ICI, prioritizing CCRT plus consolidation ICI. For patients in the mid-risk subgroup, who have a moderate short-term but poor long-term prognosis, the efficacy of consolidation ICI requires further investigation.

Our data demonstrated that longitudinal ctDNA negativity during consolidation ICI correlated with a better prognosis, while consolidation ICI provided survival benefits for patients with detectable ctDNA after chemotherapy and radiotherapy. In this study, one of six patients who achieved ctDNA clearance and maintained negativity during consolidation ICI remained alive and progression-free during a follow-up period of 33.0 months, suggesting that ctDNA clearance under consolidation immunotherapy reflects the efficacy of consolidation ICI. Although no prior study has directly investigated the relationship between ctDNA dynamics and adjuvant therapy efficacy after definitive local treatment in SCLC, similar findings have been reported in NSCLC. Zhang et al. demonstrated that patients with detectable ctDNA before adjuvant therapy who achieved early and sustained ctDNA clearance had the longest disease-free survival.^36^ Jun et al. reported that NSCLC patients with detectable ctDNA before consolidation ICI who exhibited an early ctDNA response had significantly better PFS and OS.^37^

Several limitations should be considered when our findings are interpreted. First, the relatively small sample size and limited follow-up duration, particularly in the consolidation ICI cohort and among patients with intermediate and good prognoses, may restrict the statistical power and interpretability of long-term outcomes. These constraints highlight the need for future studies with larger cohorts and prolonged follow-up to robustly validate the prognostic and predictive value of ctDNA in the context of consolidation ICI therapy. Second, the ctDNA sequencing method used in this study was a tumor-naïve approach instead of tumor-informed ctDNA detection, which potentially offers increased sensitivity for detecting residual tumors or disease progression. However, previous reports have shown that tumor-naïve ctDNA detection is comparable to tumor-informed methods in terms of ctDNA variant detection, lead time, and specificity.^12,21^ Finally, although plasma samples were prospectively collected for ctDNA detection, the retrospective nature of our analyses contributed to considerable variability in the number of samples available at each time point, and nonrandomized treatment assignment may also introduce potential biases. Nevertheless, we employed multivariable time-dependent Cox regression models adjusting for relevant confounders, and the results remained consistent, supporting the robustness of our findings.

In conclusion, our findings provide valuable insights into the individualized management of LS-SCLC in the era of immunotherapy and may guide the development of future prospective studies.

## Materials and methods

### Patients and clinical data

We performed a prospective study integrating patient data from two clinical trials evaluating different consolidation therapy regimens for LS-SCLC: CCRT alone (NCT02688036) and CCRT plus serplulimab (NCT05443646). Patients with cytologically or histologically proven LS-SCLC according to the Veterans Administration Lung Cancer Group staging system who received thoracic radiotherapy (TRT) starting at cycle 3 after commencing cisplatin/carboplatin-etoposide chemotherapy and who had ≥1 plasma ctDNA sample fulfilling the quality control requirements for sequencing were enrolled. A PCI of 25 Gy in 10 fractions was recommended for responders to CCRT. Patients in the ICI trials received consolidation serplulimab for 1 year. Post-CCRT follow-up was performed every 3 months for 2 years, every 6 months to 5 years, and every year thereafter. Demographic and clinical data were sourced from medical records. The study protocol was approved by the Ethics Committee of the National Cancer Center/National Clinical Research Center for Cancer/Cancer Hospital, Chinese Academy of Medical Sciences and Peking Union Medical College (15-068/995, 22/236-3438) and was conducted in accordance with the principles of the Declaration of Helsinki. Written informed consent was obtained from all participants before sample collection.

### Sample collection, sequencing, and ctDNA detection identification

A total of 490 plasma ctDNA samples were sequenced, comprising 251 from the CCRT-only cohort (collected between July 2018 and January 2024) and 239 from the consolidation ICI cohort (collected between July 2022 and December 2024). Plasma samples were collected at the following time points: baseline (t0), after induction chemotherapy (ICT) but before TRT (post-ICT & pre-TRT, t1), after completion of radiotherapy (post-RT, t2), including both post-TRT and post-PCI, and at progressive disease (PD, t3). For patients receiving consolidation ICI, additional samples were collected at the initiation of cycle 3 (C3), month 6 (M6), and year 1 (Y1) of ICI treatment. Only plasma samples extracted from 10 mL of peripheral blood that yielded ≥2 mL of plasma and ≥10 ng of cfDNA and passed quality control were included in downstream analysis. Plasma ctDNA was subjected to next-generation sequencing (NGS) profiling via a Pulmocan^TM^ panel covering 139 lung cancer-related genes (Supplementary Table 1) with a targeted depth of 30000x (Nanjing Geneseeq Technology Inc., Nanjing, China). Full details on sample collection, next-generation sequencing, and mutation calling are available in the Supplementary Methods. ctDNA positivity was defined using a tumor-naïve approach, where a plasma sample was considered ctDNA positive if at least one nonsynonymous somatic alteration was detected after rigorous filtering to exclude germline variants and clonal hematopoiesis of indeterminate potential (CHIP). For patients with serial plasma samples, ctDNA positivity at a given time point was determined independently on the basis of the presence of any qualifying somatic variant in that sample, regardless of whether it matched mutations identified at prior time points.

### Clinical endpoints and outcome measures

The primary endpoint was PFS, which was defined as the time interval between treatment initiation and radiological progression or death from any cause. The secondary endpoints included OS, defined as the time between treatment initiation and death from any cause. Radiological tumor responses were assessed independently via [18F]2-fluoro-2-deoxy-D-glucose positron emission computed tomography (recommended) or computed tomography with contrast according to Response Evaluation Criteria in Solid Tumors version 1.1 by one radiologist and a thoracic radiation oncologist. Locoregional progression was defined as clinical or biopsy-proven recurrence in the primary tumor or the ipsilateral hilum, mediastinum, or supraclavicular node. Distant metastasis was defined as any evidence of metastatic disease beyond the locoregional regions previously mentioned.

### Statistical analysis

Descriptive analyses were performed on the clinical characteristics of the enrolled patients. Fisher’s exact/chi-square test was used to compare the frequencies of categorical variables. Student’s t test (paired) or the Wilcoxon rank-sum test was used to test differences in continuous variables. The median follow-up time was estimated using the reverse Kaplan‒Meier method.^38^ Survival analyses were conducted using Kaplan‒Meier curves with log-rank tests. Cox proportional hazards models (with Firth’s penalized likelihood approach^39^) were fitted to estimate the hazard ratio (HR) with 95% confidence interval (CI), and the proportionality of hazards was assessed using log(−log) survival plots. Univariate Cox regression analysis was performed for each clinicopathological variable, and those with *p* values < 0.1 were subsequently included in the multivariate Cox model to adjust for potential confounders. Immortal time bias was addressed by applying a time-dependent Cox model in which consolidation ICI and PCI were treated as time-dependent covariates.^40–42^ Individuals with missing data were excluded from the analysis. All quoted *p* values were two-tailed, and *p* values < 0.05 were considered statistically significant. The data were analyzed using R software (version 4.2.2).

## Supplementary information

Supplementary Materials for Circulating tumor DNA refines consolidation immunotherapy for limited-stage small cell lung cancer patients

## Data Availability

The sequencing data are deposited in the Genome Sequence Archive (GSA) for Human under accession number HRA012744. Any additional information used in the current study is available from the corresponding author (binan_email@163.com) upon reasonable request.
