# Correction: Circulating tumor DNA refines consolidation immunotherapy for limited-stage small cell lung cancer patients

**DOI:** 10.1038/s41392-025-02526-y

**Published:** 2026-01-19

**Authors:** Yin Yang, Yuqi Wu, Jingjing Zhao, Tao Zhang, Kailun Fei, Xiaotian Zhao, Lei Deng, Zhihui Zhang, Ying Jiang, Jianyang Wang, Wenyang Liu, Xin Wang, Song Wang, Hua Bao, Xue Wu, Minyi Zhu, Qiuxiang Ou, Wei Tang, Luhua Wang, Zhijie Wang, Nan Bi

**Affiliations:** 1 Department of Radiation Oncology, National Cancer Center/National Clinical Research Center for Cancer/Cancer Hospital, Chinese Academy of Medical Sciences and Peking Union Medical College, Beijing, China; 2 State Key Laboratory of Molecular Oncology, CAMS Key Laboratory of Translational Research on Lung Cancer, Department of Medical Oncology, National Cancer Center/National Clinical Research Center for Cancer/Cancer Hospital, Chinese Academy of Medical Sciences and Peking Union Medical College, Beijing, China; 3 Geneseeq Research Institution, Nanjing Geneseeq Technology Inc., Nanjing, China; 4 Department of Radiology, National Cancer Center/National Clinical Research Center for Cancer/Cancer Hospital, Chinese Academy of Medical Sciences and Peking Union Medical College, Beijing, China; 5 Department of Radiation Oncology, National Cancer Center/National Clinical Research Center for Cancer/Cancer Hospital & Shenzhen Hospital, Chinese Academy of Medical Sciences and Peking Union Medical College, Shenzhen, China; 6 State Key Laboratory of Molecular Oncology, Department of Radiation Oncology, National Cancer Center/National Clinical Research Center for Cancer/Cancer Hospital, Chinese Academy of Medical Sciences and Peking Union Medical College, Beijing, China

**Keywords:** Predictive markers, Prognostic markers

Correction to: *Signal Transduction and Targeted Therapy* 10.1038/s41392-025-02445-y, published online 16 October 2025

After online publication of the article^1^, the authors noticed one inadvertent mistake in Acknowledgements part that needs to be corrected. The correct grant number for the “National High Level Hospital Clinical Research Funding” should be “2022-CICAMS-80102022503”.

The key findings of the article are not affected by these corrections.

The original version of this article is corrected.
