# Supplementary material for: Circulating tumor DNA refines consolidation immunotherapy for limited-stage small cell lung cancer patients
Source: Signal Transduct Target Ther. 2025 Oct 16;10:347. doi: 10.1038/s41392-025-02445-y (PMC12528726; doi:10.1038/s41392-025-02445-y)
Supplement: Supplementary file 1 — Supplementary Materials for Circulating tumor DNA refines consolidation immunotherapy for limited-stage small cell lung cancer patients [file 41392_2025_2445_MOESM1_ESM.docx]

Supplementary Materials for

Circulating tumor DNA refines consolidation immunotherapy for limited-stage small cell lung cancer patients

Yin Yang^1#^, Yuqi Wu^1#^, Jingjing Zhao^1#^, Tao Zhang^1#^, Kailun Fei^2#^, Xiaotian Zhao^3^, Lei Deng^1^, Zhihui Zhang^1^, Ying Jiang^1^, Jianyang Wang^1^, Wenyang Liu^1^, Xin Wang^1^, Song Wang^3^, Hua Bao^3^, Xue Wu^3^, Minyi Zhu^3^, Qiuxiang Ou^3^, Wei Tang^4^, Luhua Wang^5*^, Zhijie Wang^2*^, Nan Bi^6*^

Correspondence to: binan_email@163.com; jie_969@163.com; wlhwq@yahoo.com

**This PDF file includes:**

Supplementary Methods

Supplementary Figures. 1 to 8

Supplementary Table 1

**Supplementary Methods**

*Cell-free DNA extract, library preparation, and targeted panel next-generation sequencing*

Approximately 10 mL of peripheral blood was collected in ethylenediaminetetraacetic acid (EDTA) tubes and centrifuged at 1800 × g for 10 minutes at room temperature within two hours to separate plasma and leukocytes. Leukocytes were used as matched normal controls to filter germline mutations. Plasma cell-free DNA (cfDNA) was extracted from ≥2 mL of plasma using the QIAamp Circulating Nucleic Acid Kit (Qiagen Cat. No. 55114). The concentration and purity of extracted cfDNA were assessed with a Nanodrop2000 (Thermo Fisher Scientific, Waltham, MA), and cfDNA yield was quantified using the Qubit dsDNA HS Assay Kit on the Qubit 3.0 Fluorometer (Life Technologies). Samples with cfDNA yield ≥10 ng proceeded to library preparation using the KAPA Hyper Prep kit (KAPA Biosystems), which included end repair and adapter ligation performed with Agencourt AMPure XP beads (Beckman Coulter). Libraries were then amplified by polymerase chain reaction and purified. Library size distribution was evaluated using the 2100 Bioanalyzer (Agilent Technologies, Santa Clara, CA). Target enrichment was performed with customized Pulmocan^TM^ probes (Nanjing Geneseeq Technology Inc., Nanjing, China). Enriched libraries were sequenced on the Hiseq4000 platform (Illumina) targeting a depth of 30000×.

*Sequence Data Processing and Mutation Calling*

Raw FASTQ files underwent quality control using Trimmomatic^1^, where leading and trailing bases with quality scores below 20 or ambiguous (N) bases were trimmed. High-quality reads were aligned to the human reference genome (GRCh37/hg19) using modified Burrows-Wheeler Aligner (BWA-MEM, v0.7.12, https://github.com/lh3/bwa/tree/master/bwakit)^2^. PCR duplicates were removed with Picard tools (<https://broadinstitute.github.io/picard/>). Local realignment and base quality score recalibration were performed using the Genome Analysis Toolkit (GATK v4.1.4; <https://software.broadinstitute.org/gatk/>). Single nucleotide variants (SNVs) and insertions/deletions (indels) were identified using Mutect2^3^ in tumor-normal mode. Following variant calling, additional filtering steps were applied to improve specificity and remove potential artifacts, including: (1) absence from an in-house compiled panel of recurrent sequencing errors derived from 200 healthy control samples; (2) exclusion of variants with population allele frequency > 1% in public databases, including the 1000 Genomes Project, ExAC, or gnomAD; (3) variant allele frequency (VAF) ≥ 0.02% with at least two supporting reads. The final set of mutations was manually reviewed using the Integrative Genomics Viewer. Copy number variation (CNV) analysis was performed using CNVKit (v0.9.10)^4^, with depth ratios of > 2.0 and <0.6 used to define CNV gains and losses, respectively^5^.

**References**

1 Bolger, A. M., Lohse, M. & Usadel, B. Trimmomatic: a flexible trimmer for Illumina sequence data. *Bioinformatics.* **30**, 2114-2120 (2014).

2 Li, H. & Durbin, R. Fast and accurate short read alignment with Burrows-Wheeler transform. *Bioinformatics.* **25**, 1754-1760 (2009).

3 Cibulskis, K. *et al.* Sensitive detection of somatic point mutations in impure and heterogeneous cancer samples. *Nat Biotechnol.* **31**, 213-219 (2013).

4 Talevich, E., Shain, A. H., Botton, T. & Bastian, B. C. CNVkit: Genome-Wide Copy Number Detection and Visualization from Targeted DNA Sequencing. *PLoS Comput Biol* **12**, e1004873. (2016).

5 Huang, X. *et al.* Genomic profiling of advanced cervical cancer to predict response to programmed death-1 inhibitor combination therapy: a secondary analysis of the CLAP trial. *J Immunother Cancer.* **9** (2021).

**Supplementary Figure 1.**

**
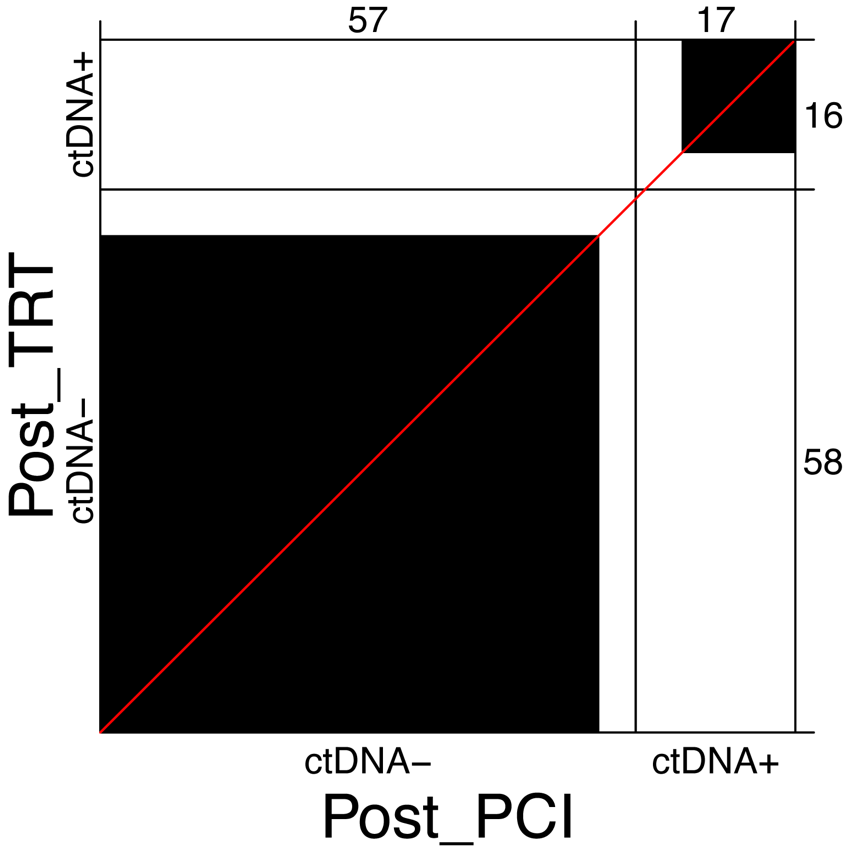
**

**Supplementary Fig. 1 Agreement plot for post-TRT and post-PCI ctDNA detection**

TRT, thoracic radiotherapy; PCI, prophylactic cranial irradiation; ctDNA, circulating tumor DNA.

**Supplementary Figure 2.**

**
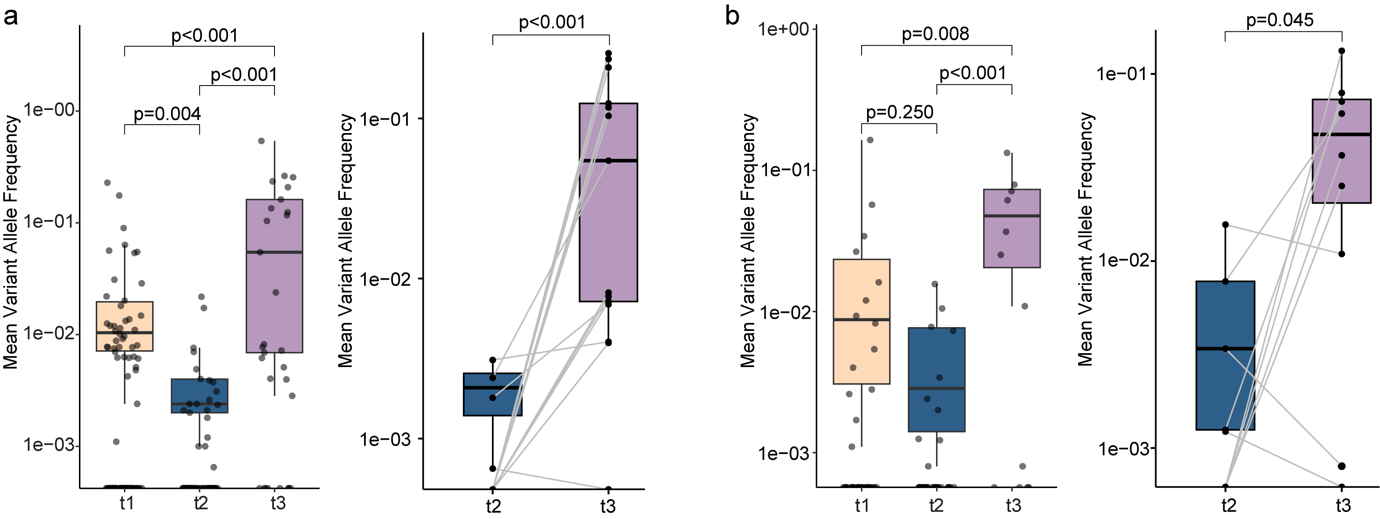
**

**Supplementary Fig. 2 Mean variant allele frequencies across time points**

**a, b,** Mean VAF changes from t1 to t3 and comparisons of paired t2 and t3 samples in patients receiving CCRT only (**a**) and CCRT followed by consolidation ICI therapy (**b**). Each dot represents the mean VAF of all detected mutations in a plasma sample at the indicated time point. VAF, variant allele frequency; CCRT, concurrent chemoradiotherapy; ICI, immune checkpoint inhibitor.

**Supplementary Figure 3.**

**
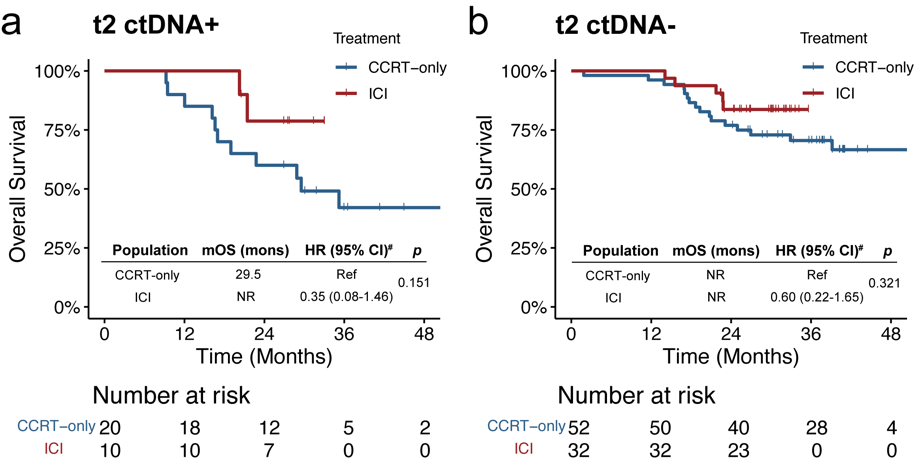
**

**Supplementary Fig. 3 Predictive value of t2 ctDNA in consolidation ICI therapy.**

**a,** Kaplan-Meier curves for OS among patients with positive t2 ctDNA status stratified by treatment regimens. **b,** Kaplan‒Meier curves for OS among patients with negative t2 ctDNA status stratified by treatment regimens. ctDNA, circulating tumor DNA; ICI, immune checkpoint inhibitor; CCRT, concurrent chemoradiotherapy; OS, overall survival; HR, hazard ratio; CI, confidence interval; NR, not reached. ^#^HR, 95% CI, and p values were estimated using time-dependent Cox regression models.

**Supplementary Figure 4.**

**
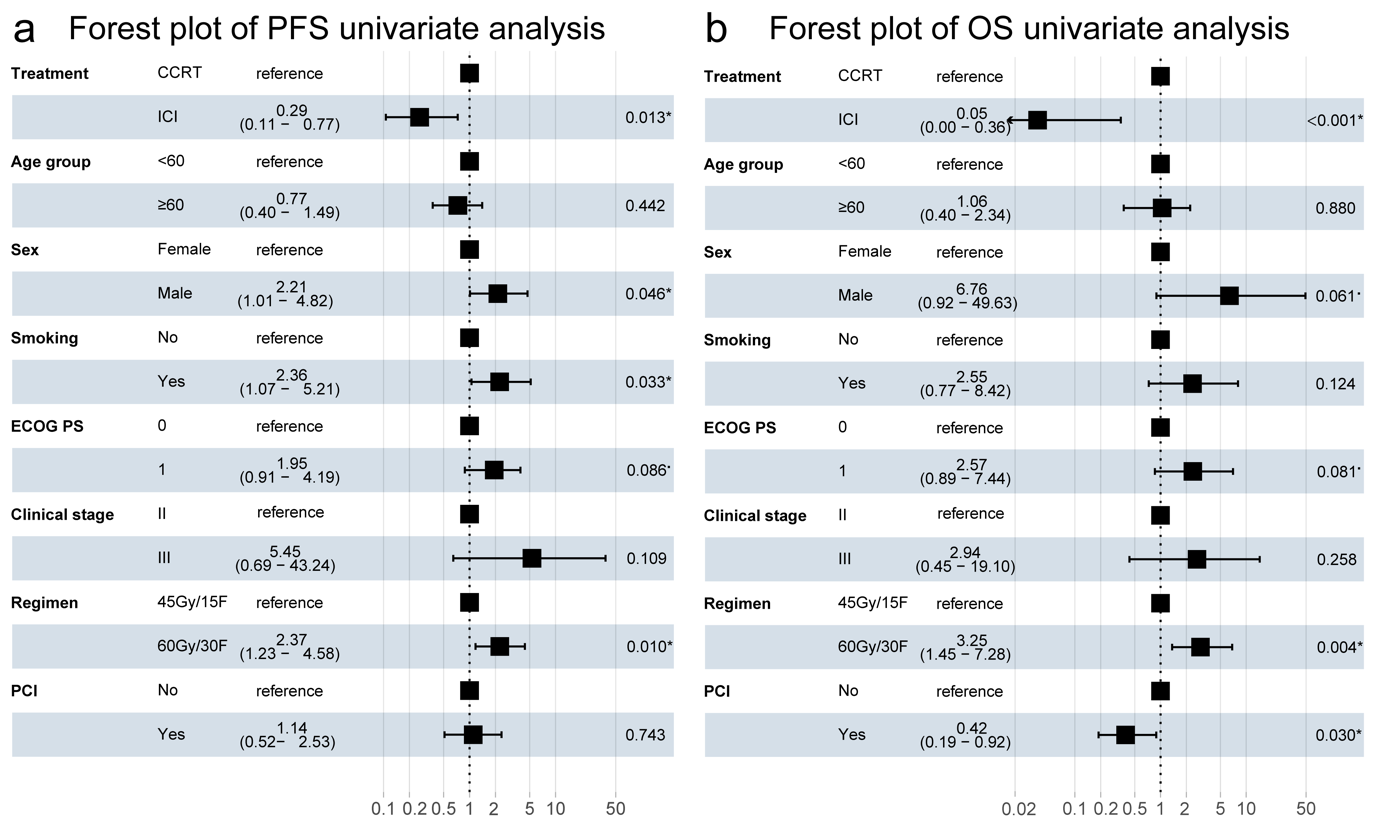
**

**Supplementary Fig. 4 Univariate time-dependent Cox regression analysis in patients with positive t1 ctDNA status.**

**a, b**, Forest plots depicting univariate time-dependent Cox regression results for progression-free survival (**a**) and overall survival (**b**). ctDNA, circulating tumor DNA; ICI, immune checkpoint inhibitor; CCRT, concurrent chemoradiotherapy; PFS, progression-free survival; OS, overall survival; ECOG PS, Eastern Cooperative Oncology Group Performance Scale; PCI, prophylactic cranial irradiation. Dots denote trend-level significance: ·p < 0.1. Asterisks indicate levels of statistical significance: *p < 0.05.

**Supplementary Figure 5.**

**
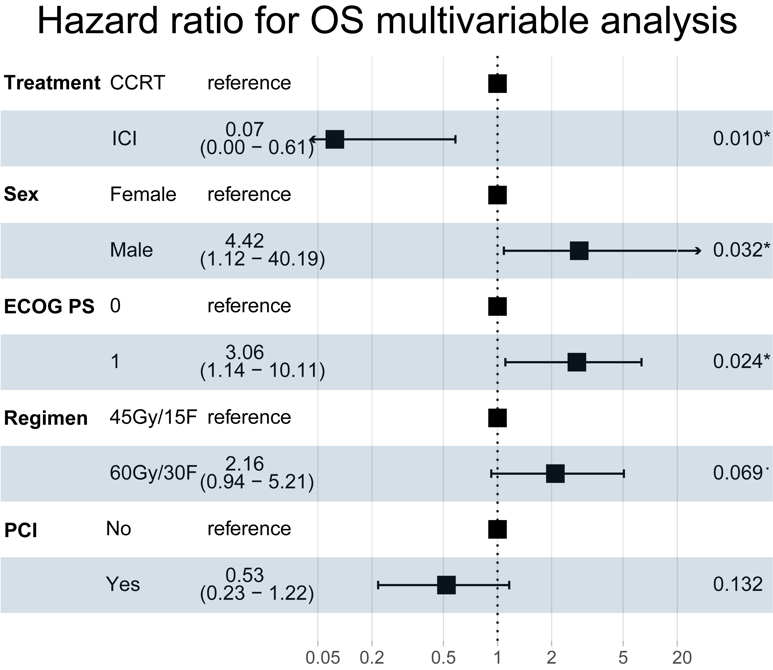
**

**Supplementary Fig. 5 Multivariate time-dependent Cox regression models for overall survival in patients with positive t1 ctDNA status**

ctDNA, circulating tumor DNA; ICI, immune checkpoint inhibitor; CCRT, concurrent chemoradiotherapy; OS, overall survival; ECOG PS, Eastern Cooperative Oncology Group Performance Scale; PCI, prophylactic cranial irradiation. ^#^HR, 95% CI, and p values were estimated using time-dependent Cox regression models. Dots denote trend-level significance: ·p < 0.1. Asterisks indicate levels of statistical significance: *p < 0.05.

**Supplementary Figure 6.**

**
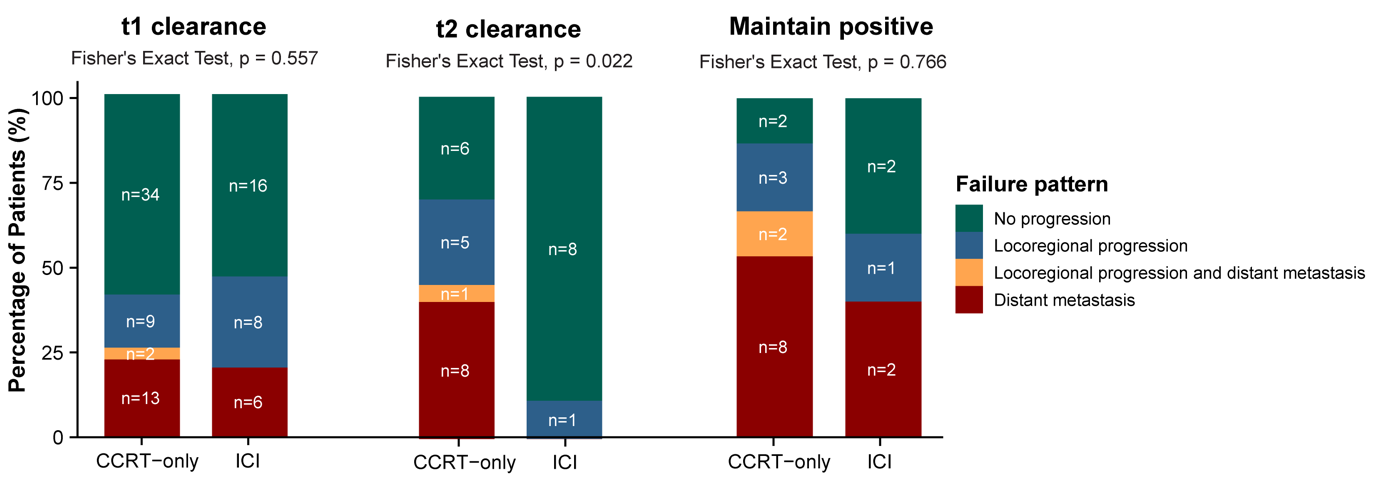
**

**Supplementary Fig. 6 Failure pattern across all subgroups**

ICI, immune checkpoint inhibitor; CCRT, concurrent chemoradiotherapy.

**Supplementary Figure 7.**

**
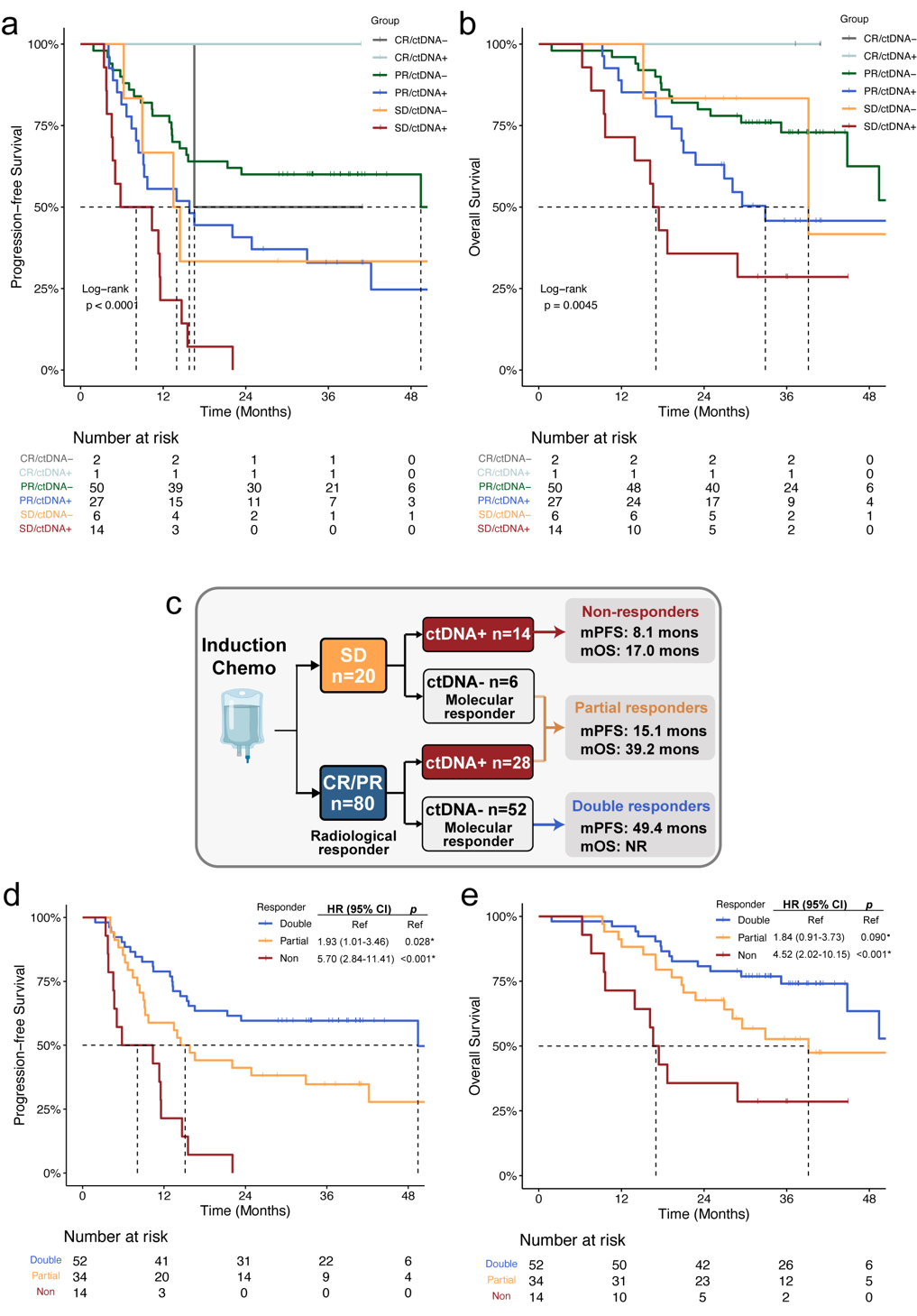
**

**Supplementary Fig. 7 Prognostic stratification by combining radiological and molecular responses in CCRT-only patients**

**a, b**, Kaplan‒Meier curves for PFS (**a**) and OS (**b**) among patients with various responses to ICT. **c**, Prognostic stratification by combining radiological and molecular responses in CCRT-only patients. **d, e**, Kaplan‒Meier curves for PFS (**d**) and OS (**e**) across double, partial, and non-responders to ICT. CCRT, concurrent chemoradiotherapy; ctDNA, circulating tumor DNA; CR, complete response; PR, partial response; SD, stable disease; PFS, progression-free survival; OS, overall survival; ICT, induction chemotherapy; HR, hazard ratio; CI, confidence interval.

**Supplementary Figure 8.**

**
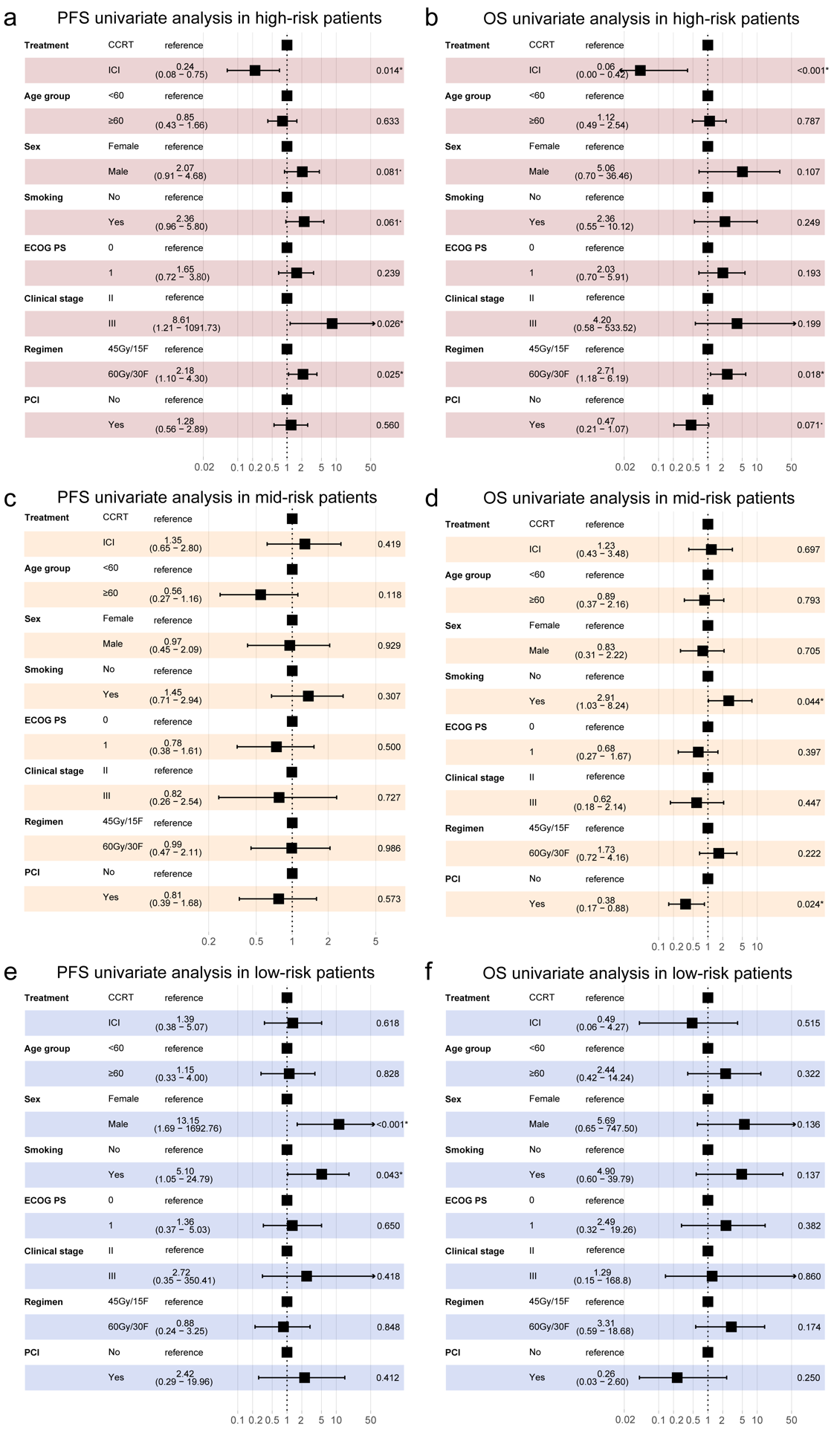
**

**Supplementary Fig. 8 Univariate time-dependent Cox regression analysis in high-, mid-, and low-risk patients**

**a, b,** Forest plots of PFS (**a**) and OS (**b**) in high-risk patients. **c, d,** Forest plots of PFS (**c**) and OS (**d**) in mid-risk patients. **e, f,** Forest plots of PFS (**e**) and OS (**f**) in mid-risk patients. PFS, progression-free survival; OS, overall survival; ICI, immune checkpoint inhibitor; CCRT, concurrent chemoradiotherapy; ECOG PS, Eastern Cooperative Oncology Group Performance Scale; PCI, prophylactic cranial irradiation. Dots denote trend-level significance: ·p < 0.1. Asterisks indicate levels of statistical significance: *p < 0.05.

**Supplementary Table 1. 139 genes covered by the next-generation sequencing panel**

| *AKT1* | *CDKN2B* | *GNAS* | *MYC* | *ROS1* |
| --- | --- | --- | --- | --- |
| *AKT2* | *CHEK2* | *GRIN2A* | *NBN* | *SBDS* |
| *AKT3* | *CREBBP* | *GSTM1* | *NF1* | *SDC4* |
| *ALK* | *CTNNB1* | *GSTP1* | *NF2* | *SETD2* |
| *APC* | *CYLD* | *GSTT1* | *NFE2L2* | *SF3B1* |
| *AR* | *CYP2B6* | *HDAC9* | *NOTCH1* | *SLC34A2* |
| *ARAF* | *CYP2C19* | *HGF* | *NQO1* | *SMAD2* |
| *ARID1A* | *CYP2D6* | *HRAS* | *NRAS* | *SMAD3* |
| *ARID2* | *CYP3A4* | *IDH1* | *NTRK1* | *SMAD4* |
| *ASXL1* | *CYP3A5* | *IDH2* | *NTRK3* | *SMARCA4* |
| *ATM* | *DDR2* | *JAK1* | *PBRM1* | *SMARCB1* |
| *ATR* | *DHFR* | *JAK2* | *PDCD1* | *SOX2* |
| *ATRX* | *DNMT3A* | *KDR* | *PDCD1LG2* | *STAG2* |
| *AXL* | *DPYD* | *KEAP1* | *PDGFRA* | *STAT3* |
| *BCL2L11* | *EGFR* | *KIT* | *PDGFRB* | *STK11* |
| *BRAF* | *ERBB2* | *KMT2A* | *PIK3CA* | *TET2* |
| *BRCA1* | *ERBB3* | *KMT2C* | *PIK3CD* | *TGFBR2* |
| *BRIP1* | *ERBB4* | *KMT2D* | *PIK3R1* | *TP53* |
| *BTK* | *ERCC1* | *KRAS* | *PTEN* | *TPMT* |
| *CD274* | *ERCC2* | *LRP1B* | *PTPN11* | *TSC1* |
| *CD74* | *ERCC4* | *LZTR1* | *QKI* | *TSC2* |
| *CDA* | *FAT1* | *MAP2K1* | *RAF1* | *TYMS* |
| *CDH1* | *FBXW7* | *MAP2K2* | *RB1* | *U2AF1* |
| *CDK4* | *FGFR1* | *MED12* | *RECQL4* | *UGT1A1* |
| *CDK6* | *FGFR3* | *MET* | *RELN* | *VEGFA* |
| *CDK8* | *FLT4* | *MLH1* | *RET* | *WRN* |
| *CDKN1B* | *FRG1* | *MTHFR* | *RHOA* | *XRCC1* |
| *CDKN2A* | *GATA4* | *MTOR* | *RICTOR* |  |
